# Establishment and characterization of mouse lines useful for endogenous protein degradation via an improved auxin‐inducible degron system (AID2)

**DOI:** 10.1111/dgd.12942

**Published:** 2024-09-21

**Authors:** Hatsune Makino‐Itou, Noriko Yamatani, Akemi Okubo, Makoto Kiso, Rieko Ajima, Masato T. Kanemaki, Yumiko Saga

**Affiliations:** ^1^ Department of Gene Function and Phenomics, National Institute of Genetics Research Organization of Information and Systems (ROIS) Mishima Japan; ^2^ Division for Development of Genetic‐Engineered Mouse Resource National Institute of Genetics Mishima Japan; ^3^ Department of Genetics Graduate Institute for Advanced Studies Mishima Japan; ^4^ Department of Chromosome Science National Institute of Genetics, ROIS Mishima Japan; ^5^ Department of Biological Sciences, Graduate School of Science The University of Tokyo Tokyo Japan

**Keywords:** Cas9 gene editing, degron, knock‐in mouse, protein knockdown, *ROSA26* locus, transgenic mouse

## Abstract

The development of new technologies opens new avenues in the research field. Gene knockout is a key method for analyzing gene function in mice. Currently, conditional gene knockout strategies are employed to examine temporal and spatial gene function. However, phenotypes are sometimes not observed because of the time required for depletion due to the long half‐life of the target proteins. Protein knockdown using an improved auxin‐inducible degron system, AID2, overcomes such difficulties owing to rapid and efficient target depletion. We observed depletion of AID‐tagged proteins within a few to several hours by a simple intraperitoneal injection of the auxin analog, 5‐Ph‐IAA, which is much shorter than the time required for target depletion using conditional gene knockout. Importantly, the loss of protein is reversible, making protein knockdown useful to measure the effects of transient loss of protein function. Here, we also established several mouse lines useful for AID2‐medicated protein knockdown, which include knock‐in mouse lines in the *ROSA26* locus; one expresses TIR1(F74G), and the other is the reporter expressing AID‐mCherry. We also established a germ‐cell‐specific TIR1 line and confirmed the protein knockdown specificity. In addition, we introduced an AID tag to an endogenous protein, DCP2 via the CAS9‐mediated gene editing method. We confirmed that the protein was effectively eliminated by TIR1(F74G), which resulted in the similar phenotype observed in knockout mouse within 20 h.

## Graphical abstract

We provide several mouse lines for a protein knockdown system useful to analyze protein function in vivo.

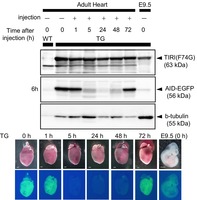

## 1 INTRODUCTION

Gene knockout technology is a key and standard method to reveal the function of corresponding genes in mice. In addition to conventional gene knockout, inducible gene knockout is possible using inducible Cre recombinase and the target sequences (Feil et al., 1996). However, as the target of this technology is the gene, there is a time lag to knockdown the protein, and the knockdown efficiency depends on the half‐life. In some cases, it may take more than 3 days to eliminate all proteins (Dhamodharan et al., 2022), which hampers functional studies, especially during embryonic stages that are associated with rapid changes in the developmental clock. Recently, several methods were developed to knockdown proteins such as the RNA interference method (Chang et al., 2004) and the degron system. As the RNA interference methods target RNA and not protein, the efficiency largely depends on the sequence used for the generation of double‐stranded RNA. In general, the knockdown is incomplete and may lead to a partial reduction of the target protein. Furthermore, the issue of off‐target effects must be considered. The latter was recently developed and is highly effective because it directly targets proteins. There are several methods to directly target proteins for degradation. Among them, we focused on the auxin‐inducible‐degron (AID) system, in which the target protein can be genetically marked by a degron tag, and reversible protein knockdown is possible with the addition of auxin (Nishimura et al., 2009). This AID system requires two components: a degron‐tagged protein and the TIR1 E3‐ligase component activated by the phytohormone auxin. This system was initially established in yeast and then demonstrated to function in cultured mammalian cells (Natsume et al., 2016; Nishimura et al., 2009). We recently improved the original AID system and established an improved system, namely AID2, by using the TIR1(F74G) mutant and the new inducer 5‐Ph‐IAA (Yesbolatova et al., 2020). The AID2 system did not exhibit leaky degradation like the original system, but induced sharp target degradation with very low inducer concentrations, and enabled reversible expression control in cell culture. Moreover, we reported that AID2 induced the degradation of an enhanced green fluorescent protein (EGFP) reporter in living mice.

Recently, a similar system, the so‐called dTAG system, was applied to the mouse system to control endogenous proteins such as NELFB (Abuhashem et al., 2022), CDK2, and CDK5 (Yenerall et al., 2023). The tagged protein was quickly removed upon administration of small molecule dTAG‐13. This system is very effective and convenient for knocking down any proteins. However, as this system relies on an endogenous proteasome pathway, proteins are ubiquitously removed from any cells. Therefore, cell‐type‐specific knockdown cannot be achieved. Whereas the AID system uses an exogenous plant‐based factor TIR1 to activate the system, it can be used for a tissue‐specific conditional knockdown system by expressing TIR1 in particular cells. In this report, we provide mouse lines established in a well‐known *ROSA26* locus, which expresses TIR1(F74G), and the reporter AID‐mCherry, which are convenient for monitoring cell‐type‐specific events. As a test case, we show that the germ‐cell‐specific TIR1 mouse line is useful to knock down AID‐tagged protein only in the germ cells. We also demonstrate that AID‐tagged endogenous protein can be quickly eliminated to manifest the phenotype.

## 2 MATERIALS AND METHODS

### 2.1 Animals

All animals were kept in a room conditioned at 23 ± 2°C, with 50 ± 10% humidity, under a 12‐hour light‐and‐dark cycle. All protocols and procedures involving the care and use of animals were reviewed and approved by the Institutional Animal Care and Use Committee of the National Institute of Genetics. Throughout the study, the care and use of animals were conducted under the guidelines and regulations set by the Ministry of Education, Culture, Sports, Science and Technology, the Ministry of the Environment, and the Science Council of Japan. The mouse lines used for the production (B6/C3H‐F1), maintenance (C57BL/6), and embryonic analyses (MCH) were purchased from CLEA Japan, Inc. (Tokyo, Japan).

### 2.2 Generation of transgenic mice

The method for producing transgenic (TG) mouse lines pMK411#13, #16, and #19 was described previously (Yesbolatova et al., 2020). Briefly, the plasmid DNA fragment containing *CAG‐TIR1‐p2a‐AID‐EGFP‐luk* was injected into the pronucleus of fertilized eggs (B6C3F1). TG mice were identified by polymerase chain reaction (PCR) with GFP‐L1 and GFP‐R1 primers. A similar strategy was used to establish a TIR1‐reporter mouse line, TG‐CAG‐AID‐mCherry. The DNA construct is composed of a CAG promoter‐enhancer followed by *AID‐tagged mCherry* (*mCherry* was originally purchased from Clontech) and a rabbit β‐globin poly A‐signal sequence.

To produce knock‐in mice in the *ROSA26* locus, the Cas9‐mediated gene‐editing method was employed. A targeting vector is composed of *ROSA26* 5′ (802 bp) and 3′ (1052 bp) genomic sequences flanking the *CAG‐AID‐mCherry* construct. The single‐stranded targeting vector was generated using the TAKARA Guide‐it™ Long ssDNA Production System (632644). The single‐stranded DNA was injected with CRISPR‐Cas9 gRNA complex (IDT) and TrueCut Cas9 protein v2 (Invitrogen) into the pronucleus of fertilized eggs (B6C3F1). We used the ES‐mediated method to introduce *CAG‐TIR1(F74G)‐3xFlag* into the *ROSA26* locus. The targeting vector composed of *ROSA26* 5′ (802 bp) and 3′ (1052 bp) flanking the *CAG‐TIR1‐3xFag* and the pX330 (Addgene, #42230) vector containing a guide RNA to the *ROSA26* locus and PGK‐puro were transfected to ES cells (derived from B6J and established in‐house). Correctly recombined clones were used to generate chimera mice.

To produce a germ‐cell‐specific TIR1‐expressing TG mouse line, a DNA construct composed of *Oct4* enhancer lacking the proximal element (Yoshimizu et al., 1999) followed by *TIR1* (*F74G*) with *3x‐Flag* tag and poly‐A signal was injected into the fertilized egg.

To produce the AID‐tagged DCP‐2 mouse line, a targeting vector was constructed as follows. To introduce the AID tag just after the ATG translation star codon, a DNA cassette containing AID sequences (204 bp for 68 amino acids) was inserted in frame in exon‐1. The targeting vector comprised a 5′ UTR (645 bp) region, a part of exon‐1, and a part of exon‐1 and intron‐1 (632 bp) as left and right arms, respectively. The single‐stranded DNA was injected with single guide RNA (IDT) and TrueCut Cas9 protein v2 (Invitrogen) into the pronucleus of fertilized eggs (B6C3F1). A DCP2‐KO mouse line was also established via CAS9‐mediated gene editing by injecting two single guide RNA (DCP2‐site1 and site2, located in intron1 and intron2, respectively) and Cas9 protein into fertilized eggs. All primer and Cas9 target site information is shown in Table S1.

Features of the mouse lines produced are described in Table S2.

### 2.3 Inverse‐PCR to identify integration sites of pMK411‐#16 and #19

One microgram of genomic DNA prepared from pMK411#16 and #19 was digested with *Hae*III and self‐ligated with T4 ligase (TAKARA, 2011A). After purification, PCR amplification was carried out using primers pMK411 *Hae*III Fw1: 5′‐*ATAATTTTGTTGCCGTTCCACAG*‐3′ and pMK411 *Hae*III Rv1: 5′‐*TGCATTCTAGTTGTGGTTTGTCC*‐3′ or pMK411 *Hae*III Rv2: 5′‐*GCCATACCACATTTGTAGAGGTTTT*‐3′. Clear PCR bands generated by the specific primers were sequenced and integration sites were identified. Primers used for genotyping are shown in Table S1.

### 2.4 Protein knockdown experiment

To induce protein degradation, 5‐Ph‐IAA (BioAkademia, Japan, #30‐003) dissolved in phosphate‐buffered slaine (PBS; 0.5 mg/mL) was injected intraperitoneally (final concentration was 5 mg/kg unless indicated). Images of tissues and embryos were taken on a LEICA MZ16F stereomicroscope equipped with an OLYMPUS DP74 camera.

### 2.5 Quantification method of fluorescence signals

EGFP and mCherry intensities were measured by using ImageJ software as described previously (Yesbolatova et al., 2020). Briefly, fluorescence images were converted to 8‐bit grayscale images. The embryo outlines were selected and the measured mean value was recorded. The background intensity was also measured outside the embryo and the value was subtracted.

### 2.6 Western blotting analysis

Tissue or embryo samples were immediately frozen in liquid nitrogen. For electrophoresis, frozen samples were lysed in TNE buffer (50 mM Tris–HCl pH 7.4, 150 mM NaCl, 1 mM dithiothreitol, 1 mM ethylenediamine tetraacetic acid, and 1% Nonidet‐P40) with cOmplete Protease inhibitor cocktail (Roche). After centrifugation, the protein concentration of supernatants was measured with Protein Assay Dye Reagent Concentrate (Bio‐Rad). Appropriate amounts were mixed with 2× SDS sample buffer (Tris–HCl pH 6.8, 4% sodium dodecyl sulfate (SDS), 20% glycerol, 10% 2‐mercaptoethanol, and 0.004% bromophenol blue) and incubated at 95°C for 5 min before loading. After electrophoresis, proteins were transferred onto an Immobilon‐P Transfer Membrane (Millipore). The membrane was incubated with a primary antibody in skim milk or in Can Get Signal (TOYOBO, NKB101) at 4°C overnight and subsequently incubated with a secondary antibody at room temperature for 2–3 h. Detection was performed using the SuperSignal West Femto Maximum Sensitivity Substrate (Thermo Scientific) and images were acquired with a ChemiDoc Touch MP system (Bio‐Rad).

### 2.7 Antibodies used for Western blot analyses

For protein detection, the following commercially available antibodies were used at the indicated concentrations. Primary antibodies: anti‐OsTIR1 (1:1000, MBL, PD048), anti‐mAID (1:1500, MBL, M214‐3), anti‐DCP2 (1:1000, Sigma‐Aldrich, ABE2901), anti‐mCherry (1:2000, Thermo Fisher, M11217) anti‐β‐tubulin (1:3000, Sigma‐Aldrich, T4026), and anti‐β‐actin (1:5000, Sigma, A5441). For secondary antibodies, anti‐rabbit IgG horseradish peroxidase (HRP) (Cell Signaling, 7074S), anti‐mouse IgG HRP (Cell Signaling, 7076S), and anti‐rat IgG HRP (Abcam, ab97057) were used at a 1:5000 dilution. To detect Flag‐tagged TIR1, anti‐FLAG‐M2‐HRP (Sigma‐Aldrich, A8592) was used at a 1:5000 dilution.

### 2.8 Immunofluorescence staining

Embryonic testes and brains were fixed in 4% paraformaldehyde on ice for 1 h. Samples were treated with 10%, 20%, and 30% sucrose sequentially and embedded in OCT compound (Tissue Tek, Sakura), and frozen. The 6‐μm frozen sections were incubated with 3% skim milk for 1 h and subjected to the primary antibodies overnight at 4°C. After washing with PBST (PBS containing 0.1% Tween), sections were incubated with secondary antibodies containing bisbenzimide H33342 for 1–2 h at room temperature. After washing with PBST, the sections were mounted and observed with an Olympus FV1200 or FV3000 confocal microscope. Primary antibodies were used at the following dilutions: goat anti‐E‐cadherin (1:400, R&D Systems, AF748), rat anti‐mCherry monoclonal (1:200, Thermo Fisher, M11217), mouse anti‐FLAG M2‐peroxidase (HRP) (1:1000, Sigma‐Aldrich, A8592), and rabbit anti‐DDX6 (1:300, Bethyl, A300460A). For secondary antibodies, donkey anti‐rabbit, anti‐goat, and anti‐rat antibodies, conjugated with either Alexa‐488, Alexa‐594, or Alexa‐647, were used at a dilution of 1:1000. FLAG signal was detected by the HRP conjugated with fluorescein isothiocyanate via TSA‐Plus Fluorescein System (PerkinElmer).

## 3 RESULTS

### 3.1 Further characterization of TIR1‐expressing transgenic mouse lines with AID‐tagged reporter

As reported previously, we generated TG mouse lines using a construct called pMK411:*CAG‐TIR1‐p2a‐AID‐EGFP‐luk*, which expresses both the TIR1(F74G) E3‐ligase required for protein degradation and the target AID‐tagged EGFP‐luciferase reporter (Yesbolatova et al., 2020). We established three independent TG lines, pMK411#13, #16, and #19 (#13 and #16 were described before, Table S2). As shown in Figure S1a, the expression level varied among organs, but the organ specificity among lines was relatively similar. Lines #13 and #19 expressed higher levels of the reporter than #16. As line #13 exhibited lower reproductivity, we terminated this line and used #16 and #19 for further detailed characterization. In each organ, we detected both TIR1(F74G) and AID‐EGFP by Western blotting, indicating that both proteins were transcribed together (Figure S1b). However, the relative amounts of these two proteins were not always similar, suggesting different protein stabilities in each organ. The expression levels were higher in #19 than in #16. Reverse PCR identified the integration site for #16 and #19 at a different locus in chromosome 10 (Figure S2). The different expression level may be a result of the integration site and/or copy number integrated.

**FIGURE 1:**
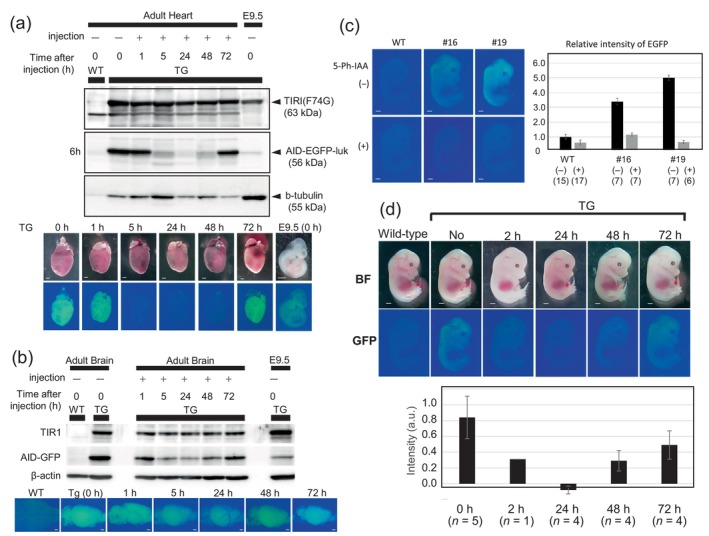
Reversible protein knockdown in adult organs and developing embryos. (a, b) Western blot analyses showed reduction and recovery of AID‐EGFP in the heart (a) and brain (b) upon administration of 5‐Ph‐IAA (5 mg/kg). Samples were prepared 1, 5, 24, 48, and 72 h after injection into adult pMK411#19 mice. An E9.5 embryo sample of pMK411#19 was used as a loading control. The same amount of protein (30 μg) was loaded. Each picture was taken before sample preparation. Scale bar, 1 mm. (c) Comparison of two transgenic (Tg) lines, pMK411#16 and #19. Embryos recovered from pregnant mothers crossed with each male Tg with (+) and without (−) injection of 5‐Ph‐IAA (5 mg/kg). EGFP reporter intensities were measured 1 h after injection and the intensities were compared. Scale bar, 1 mm. (d) Protein knockdown and recovery in developing embryos visualized by EGFP expression. 5‐Ph‐IAA (1 mg/kg) was injected into pregnant MCH mothers crossed with pMK411#16 at E10.5 (for 72 h), E11.5 (for 48 h), E12.5 (for 24 h), and E13.5 (for 2 h), and all embryos were examined at E13.5. Scale bar, 1 mm. Quantification data are shown as a bar graph. Mean intensities were calculated by subtracting the values of control (Tg‐negative) embryos in each litter.

As previously reported, the reporter protein AID‐EGFP was quickly degraded within 6 h after 5‐Ph‐IAA injection to adult mice at a concentration of 5 mg/kg (Yesbolatova et al., 2020, Figure 1a). As the re‐expression of an EGFP reporter occurred within a few hours after washing out the ligand from the cell culture, we asked how long it took for re‐expression in mice. Because we could not remove the ligand in vivo, the protein recovery rate depended on the clearance rate of the ligand in each organ. We examined the adult brain and heart because they may have different efficiencies due to the different rates of tissue penetration and/or clearance speeds (Yesbolatova et al., 2020). Protein degradation was monitored by both EGFP fluorescence and Western blotting. In the case of the heart, the EGFP signal disappeared after 6 h and the protein was at a minimum until 24 h, but recovered after 48 h, and returned to the original level by 72 h (Figure 1a). In the case of the brain, the efficiency of protein degradation was low because of inefficient ligand distribution as a result of the blood–brain barrier (Yesbolatova et al., 2020; Puris et al., 2022), nevertheless, the protein level decreased until 24 h, started to recover thereafter, and had returned to the normal level at 72 h after injection (Figure 1b). Hence, although the protein degradation rate was low in the case of the brain, the recovery speed was similar to that in the heart. In either case, TIR1(F74G) expression was maintained at a constant level, as expected.

We also tested whether the protein knockdown could be induced ex vivo. We removed the brain and colon, and incubated them in the presence of 5‐Ph‐IAA at 0.5 μM, an effective dosage to induce degradation in cultured cells (Yesbolatova et al., 2020). However, no reduction in reporter signal was observed even after 4 h (Figure S3). Therefore, we concluded that the ligand must be delivered via the body circulation system for protein knockdown in organs.

### 3.2 Protein degradation induced in embryonic stages via the umbilical cord

Next, we examined the expression levels of the EGFP reporter in the embryonic stages. Based on the fluorescence at E13.5, EGFP was ubiquitously expressed in the whole body in both #16 and #19. Stronger fluorescence was observed in the neural tube in #19, although no major difference was noted in other tissues. Quantitative analyses demonstrated approximately two‐fold stronger expression in #19 than in #16 (Figure 1c). We also measured the EGFP level after injecting 5‐Ph‐IAA into the pregnant mother, and the reporter signal diminished by up to 85% and 95% 1 h after injection (Figure 1c). Using the #16 line, we conducted a recovery assay. We first determined the minimum dosage required for reporter depletion by injecting different amounts of 5‐Ph‐IAA (0.04, 0.2, 1, and 5 mg/kg) into the pregnant mother harboring E13.5 embryos. Then, we monitored EGFP intensity in the embryos 2 h after injection. No EGFP‐positive embryos were recovered among the PCR‐positive ones when we injected more than 1 mg/kg, whereas one of seven PCR‐positive embryos exhibited residual signal at 0.2 mg/kg and all PCR‐positive embryos exhibited EGFP signal after 0.04 mg/kg injection (Figure S3b). Therefore, we judged that 1 mg/kg injection into the pregnant mother was sufficient to induce effective protein degradation in the embryos. Using this dosage, we conducted the recovery experiment. As embryos develop quickly and are difficult to monitor for longer periods, we adjusted the final observation point to E13.5, and injected 5‐Ph‐IAA at E10.5–E12.5 to monitor 24‐ to 72‐h periods. The time course of recovery was similar to that in the adult case. The EGFP signals decreased to a minimum after 24 h, then recovered gradually and returned to a considerable level after 72 h (Figure 1d), indicating that the 5‐Ph‐IAA was almost removed from the mother's body within 72 h in mice.

### 3.3 Establishment of AID‐tagged mCherry reporter lines

**FIGURE 2:**
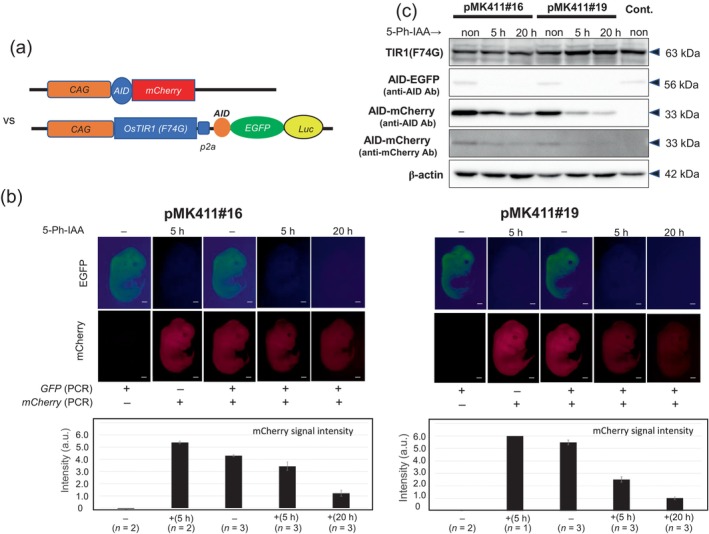
Characterization of a TIR1‐reporter line, TG‐CAG‐AID‐mCherry. (a) A male TG‐CAG‐AID‐mCherry mouse was crossed with pMK411#16 or #19 females. (b) Fluorescence signals of EGFP and mCherry were examined in E13.5 embryos from pregnant mothers with or without administration of 5‐Ph‐IAA (5 mg/kg) for 5 or 20 h. The genotype of each embryo (shown as + or –) was determined by polymerase chain reaction (PCR) after imaging. Scale bar, 1 mm. Quantitative data of fluorescent signals are shown below pictures. mCherry intensities were calculated using a similar method to Figure 1(d). (c) Western blotting analyses of transgene expression. TIR1(F74G) was detected by an anti‐TIR1 antibody. AID‐EGFP was detected by an anti‐AID antibody. AID‐mCherry signal was analyzed by anti‐AID and anti‐mCherry antibodies. β‐actin was an internal control. The expected protein size is indicated on the left side of the gel. pMK411#19 without crossing with TG‐CAG‐AID‐mCherry was used as a control.

Although the pMK411 mouse lines are useful to monitor protein knockdown events quickly in multiple organs, we need a reporter mouse line to characterize tissue‐specific TIR1 lines for more specific protein knockdown experiments. For this purpose, we developed TG mouse lines harboring *AID‐tagged mCherry* under the control of a CAG promoter‐enhancer (Figure 2a). We obtained three independent F1 lines exhibiting different levels of mCherry expression (Figure S4a). The embryo showed ubiquitous expression, and stronger expression was observed in the heart, brain, and thymus (Figure S4b). Subsequently, we crossed these reporter lines with pMK411#19 (Figure S4a) and injected 5‐Ph‐IAA into the pregnant mother at E12.5 to examine EGFP and mCherry expression in the embryos at E13.5 (after 20 h). Although the intensity of mCherry differed among the three lines, the mCherry signal was reduced in the presence of TIR1(F74G) expressed from the pMK411 transgene (Figure S4a). We selected Type‐III and crossed pMK411#16 and #19 to ask whether we could evaluate TIR1 activity using this reporter line (Figure 2b,c). In either case, we never observed the EGFP signal 5 h after 5‐Ph‐IAA injection (Figure 2b), indicating that TIR1 expressed from #16 and #19 was sufficient to degrade AID‐EGFP even in the presence of AID‐mCherry. However, we observed that mCherry intensity differed between #16 and #19. Fluorescence quantification results indicated that the mCherry signal was reduced by 54% via #19, but only 20% reduction was achieved via #16 after 5 h (Figure 2b). These results were confirmed by Western blot analyses (Figure 2c). AID‐mCherry protein detected by anti‐AID antibody was sharply reduced in pMK411#19 compared with #16, which may be correlated with the expression level of TIR1(F74G).

### 3.4 Establishment of independent mouse lines expressing TIR1(F74G) or the AID‐mCherry reporter from *ROSA26* locus

**FIGURE 3:**
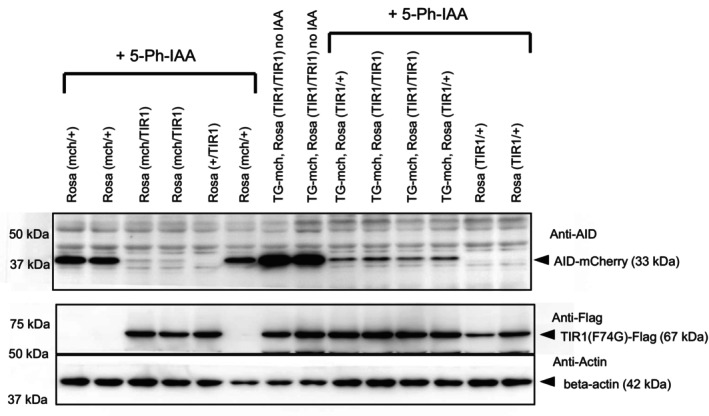
The effect of TIR1 derived from the *ROSA26* locus on AID‐mCherry reporters. Western blotting analyses of E13.5 embryonic brains expressing either TG‐AID‐mCherry (shown as TG‐mch) or Rosa‐AID‐mCherry (shown as Rosa (mch/+)) crossed with Rosa‐TIR1. Rosa(mch/TIR1) indicates double heterozygous. 5‐Ph‐IAA (5 mg/kg) was injected into the E12.5 pregnant mothers and the embryos were recovered at E13.5. The genotype of each embryo is indicated at the top of each lane. The AID‐mCherry signal was analyzed by anti‐AID antibody. β‐Actin was an internal control. The expected protein size is indicated on the left side of the gel.

As the TG lines we established showed variable expression levels because of the integration site and the copy number, we generated knock‐in mice in a well‐known and generally used *ROSA26* locus. The backbone DNAs were similar to those used for TG shown in Figure 2a. For TIR1(F74G) line, we removed *p2a AID‐EGFP‐Luc* and replaced it with *3xFLAG‐tag* (Figure S5). *CAG‐TIR1*(*F74G*) was introduced via the ES‐mediated method (Figure S5), while *CAG‐AID‐mCherry* was directly inserted via microinjection (Figure S6). Successful recombination was confirmed by genomic PCR. To compare the reporter activity between TG‐CAG‐AID‐mCherry and Rosa‐CAG‐AID‐mCherry and to measure the activity of the Rosa‐CAG‐TIR1‐3xFLAG line, we compared protein expression levels between Rosa‐CAG‐AID‐mCherry and TG‐CAG‐AID‐mCherry crossed with Rosa‐CAG‐TIR1(F74G). We injected 5‐Ph‐IAA into E12.5 pregnant female mice and prepared E13.5 embryonic brains for Western blot analysis (Figure 3). We observed stronger AID‐mCherry expression in TG‐CAG‐mCherry line compared with those from *ROSA26* locus (Figure 3), probably because of the multiple transgene integration in the TG line. Those signals were strongly reduced after 5‐Ph‐IAA injection in the presence of Rosa‐TIR1(F74G) expression revealed by reactivity with anti‐Flag antibody. However, a considerable amount of AID‐mCherry remained in the case of TG‐AID‐mCherry even in the presence of two copies of Rosa‐TIR1(F74G), indicating that TIR1(F74G) expressed from the *ROSA26* locus was not strong enough to degrade a large amount of AID‐tagged target protein.

### 3.5 Establishment of a germ‐cell‐specific TIR1(F74G)‐expressing mouse line

**FIGURE 4:**
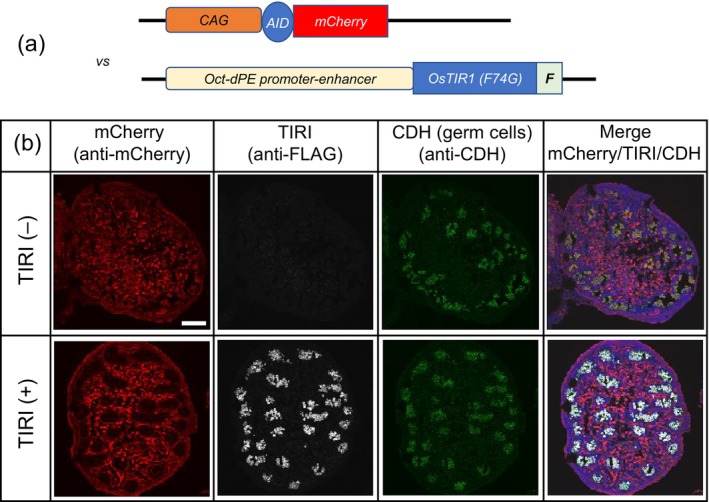
The activity of the germ cell‐specific TIR1 line. (a) The germ cell‐specific TIR1 line was crossed with the reporter line TG‐CAG‐AID‐mCherry. (b) Immunofluorescence images of embryonic testes (E15.5) prepared from a pregnant CAG‐AID‐mCherry mother crossed with an Oct‐dPE‐TIR1(F74G)‐FLAG male. TIR1(−) indicates sample without Oct‐dPE‐TIR1(F74G) and TIR1(+) indicates double transgenic sample. The mother received 5‐Ph‐IAA by injection at E14.5. Antibodies used were anti‐mCherry, anti‐FLAG, and anti‐E‐cadherin (CDH). The scale bar indicates 100 μm.

As a test case of establishing a cell‐type‐specific protein knockdown system, we aimed to express TIR1(F74G) under the control of an Oct‐dPE‐promoter‐enhancer known to be active only in germ cells from the embryonic stage (Yoshimizu et al., 1999). We established a TG line harboring *Oct‐dPE* driven *TIR1(F74G)‐Flag* and crossed it with a TG‐AID‐mCherry line (Figure 4a). We injected 5‐Ph‐IAA into a pregnant mother carrying E14.5 embryos. Subsequently, embryonic testes were removed at E15.5 to prepare frozen sections. The sections were then subjected to immunofluorescence staining to visualize the TG‐AID‐mCherry reporter and TIR1(F74G). We also used anti‐E‐cadherin to visualize germ cells. As shown in Figure 4b, the mCherry signal disappeared only from germ cells, indicating that TIR1(F74G) expressed in the germ cells induced degradation of the AID‐mCherry reporter protein.

### 3.6 The endogenously tagged protein was effectively degraded via the AID2 system

**FIGURE 5:**
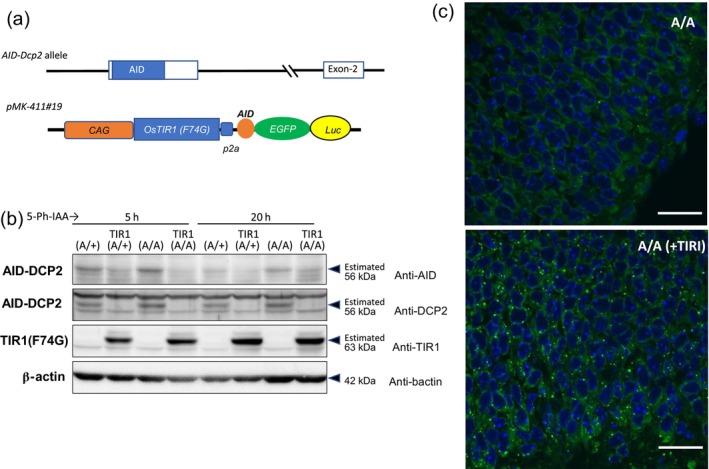
Endogenous AID‐tagged protein can be degraded quickly in vivo. (a) The homozygous AID‐DCP2 female mouse was crossed with an AID‐DCP2/pMK411‐#19 double heterozygous male. (b) Western blot analyses of E15.5 brain lysate for the detection of AID‐tagged DCP2 by anti‐AID and anti‐DCP2 antibodies. Pregnant female mice received intraperitoneal administration of 5‐Ph‐IAA (5 mg/kg) at E14.5 (for 20‐h treatment) and E15.5 (for 5‐h treatment). Genotype was indicated on each gel line. A/A indicates homozygous AID‐DCP2, A/+ indicates heterozygous AID‐DCP2. TIR1 indicates the presence of pMK411‐#19 allele. (c) Immunofluorescence images of embryonic brains treated with 5‐Ph‐IAA for 20 h (same sample used as for (b)). P‐body signals were detected by anti‐DDX6 (green). A/A indicates homozygous AID‐DCP2, and A/A(+TIR1) indicates homozygous AID‐DCP2 including pMK411#19. Nuclear staining was conducted using Hoechst 33342 (blue). The scale bar indicates 20 μm.

Based on the efficacy of the AID system, we generated knock‐in mice aiming to control endogenous proteins. The gene we targeted was the *Dcp2* gene, known to be involved in RNA degradation and expressed ubiquitously in the embryonic stage (Van Dijk et al., 2002). We generated a targeting vector to introduce the AID‐tag at the N‐terminal of DCP2 (Figure S7). The heterozygous mice were intercrossed, resulting in the production of viable homozygous AID‐DCP2 mice. As homozygous DCP2‐null mice were reported to die in the embryonic stage (Li et al., 2020), AID‐tagged DCP‐2 protein is functional. Initially, we established AID‐DCP2 and pMK411‐#19 double heterozygous mice. Subsequently, the male mice were crossed with homozygous AID‐DCP2 female mice to obtain embryos harboring AID‐DCP2 in both alleles. We injected 5‐Ph‐IAA into the pregnant mother carrying E14.5–15.5 embryos and recovered the embryos 5 or 20 h after injection. The brain samples were subjected to Western blotting analysis. As shown in Figure 5b, AID‐tagged DCP2 was detected using both anti‐AID and anti‐DCP2 antibodies, although anti‐AID antibody did not react strongly (Figure 5b). The expression level of AID‐DCP2 was higher in the homozygous embryo (A/A) than in the heterozygous (A/+) one. Importantly, the AID‐tagged protein disappeared in the embryo containing the pMK411 transgene (marked as TIR1) even at 5 h after treatment from both heterozygous and homozygous embryos. Therefore, the TIR1(F74G) expressed from the pMK411 transgene can effectively target endogenous protein. To examine possible phenotypes upon DCP2 knockdown, we prepared frozen sections from the same brain samples analyzed by Western blotting (20 h after the 5‐Ph‐IAA injection) and conducted immunohistochemistry. Loss of DCP‐2 results in P‐body enlargement (Aizer et al., 2014; Teixeira & Parker, 2007); therefore, we used an anti‐DDX6 antibody to visualize P‐bodies. We also confirmed that DDX6‐positive P‐body enlargement in the DCP2‐null embryo (Figure S8). As shown in Figure 5c, the number and size of P‐bodies increased in the TIR1‐expressing homozygous AID‐DCP2 brain compared with the control lacking TIR1(F74G), confirming that protein knockdown of DCP2 via an AID‐mediated system was effective to induce the knockout phenotype within 20 h.

## 4 DISCUSSION

We previously reported the production of mouse lines useful for protein knockdown (Yesbolatova et al., 2020). This report is for the further characterization of the mouse model and the additional mouse lines useful to conduct protein knockdown experiments. In addition, we demonstrated that endogenously tagged proteins can be degraded in vivo.

We confirmed that the ligand 5‐Ph‐IAA had no toxicity even at a higher concentration of up to 10 mg/kg, which is 10 times higher than the effective dose we used in vivo (Yesbolatova et al., 2020). However, the brain signal did not disappear even at this higher concentration, probably because of the blood–brain barrier (Puris et al., 2022). An additional invention is required to overcome this problem. In other tissues and developing embryos including the brain, protein depletion was quick, and the protein level was retained at an undetectable level until 24 h after one injection. However, the recovery was rather slow and took 3 days to return to the original protein level. Therefore, the reversibility of protein knockdown was confirmed, but additional competitors or antagonists need to be developed to achieve quicker recovery.

As this system is highly efficient in both cultured cells and living organisms (Bates et al., 2021; Hatoyama et al., 2021; Hills‐Muckey et al., 2022; Natsume et al., 2016; Negishi et al., 2022; Nishimura et al., 2009; Trost et al., 2016; Zhang et al., 2015), we expected that protein knockdown might be induced by exposing the organs to medium containing 5‐Ph‐IAA even outside the body. However, this was not the case. Therefore, the circulatory system is essential to deliver the ligand to each organ.

We also noticed that protein degradation was rapid in the embryonic stage as early as 1 h after injection. As we injected 5‐Ph‐IAA into pregnant mothers intraperitoneally, the ligand must be quickly absorbed and circulated to embryos via the umbilical cord. This quick effect is beneficial for developmental biologists who want to observe a quick response upon loss of protein function.

The AID2 system requires at least two mouse lines: an AID‐tagged line and a TIR1(F74G)‐expressing line, which is similar to the conditional gene knockout system that requires a floxed gene allele and an appropriate Cre‐expressing line. However, compared with the cKO system, in which at least 1–3 days are needed to observe the disappearance of protein and the immediate effects of protein depletion are difficult to detect, the quick knockdown of proteins via the AID2 system is highly advantageous for functional analyses (Kanemaki, 2022). A gradual decrease in protein may lead to misunderstanding of the protein function. As a test case, we generated an AID‐tagged DCP2 mouse line. Although it was suggested that the loss of DCP2 leads to P‐body enlargement in yeast and cultured cells, the consequence in living bodies had not been previously addressed. Initially, we generated a DCP2‐KO mouse line, which was embryonic lethal before E12.5 as reported previously. Nevertheless, we found enlarged DDX6‐positive P‐bodies in the knockout embryo. DCP2 is known to target many mRNAs localized in the P‐body (Luo et al., 2020). The lack of DCP2 resulted in the accumulation of unprocessed transcripts, which would be the cause of P‐body enlargement, as suggested. Importantly, a similar phenotype was observed in the embryonic brain of the AID‐DCP2 mouse just 20 h after the 5‐Ph‐IAA injection, indicating that this phenotype is an immediate response induced by the lack of DCP2. Therefore, this AID system is useful to examine protein function with sharp timing regulation.

## AUTHOR CONTRIBUTIONS

YS and MKanemaki designed the experiments. HI, NY, AO, RA, MKiso, and YS performed mouse production and the experiments using those mouse lines. HI, RA, and YS analyzed the data. YS wrote the paper.

## Supporting information


**Figure S1.** Characterization of three independent transgenic mouse lines. (a) Adult mice of three independent lines, pMK411#13, #16, and #19, were dissected and the EGFP fluorescence of each organ was recorded. A C57BL/6J mouse was used as a negative control. The exposure and imaging conditions were the same. Scale bars, 1 mm. (b) The expression levels of transgenes in indicated organs were compared between pMK411#16 and #19 using anti‐TIR1, AID, and β‐actin antibodies. β‐actin expression was undetectable in the heart and quadriceps muscles.
**Figure S2.** The integration sites of the transgene in pMK411#16 and #19. Both transgenes were inserted into chromosome 10 at different sites. Sequences around the insertion site and primer sets for genotyping are shown.
**Figure S3.** Protein knockdown outside the body. (a) Parts of the adult brain and colon dissected from pMK411#13 were incubated in the medium with and without 5‐Ph‐IAA. The reporter EGFP signal was photographed every 1 h for up to 4 h. (b) EGFP reporter intensities of embryos were measured 2 h after 5‐Ph‐IAA injection at different concentrations (0–5.0 mg/kg) into pregnant MCH mothers crossed with pMK411#16 at E13.5. Scale bar, 1 mm.
**Figure S4.** Characterization of transgenic mouse lines harboring *CAG‐AID‐mCherry*. (a) Three distinct TG lines (Type I–III) were established from one transgenic mouse (F0). Those lines were crossed with pMK411#19. The pregnant mother was injected with a single dose of 5‐Ph‐IAA (5 mg/kg) at E12.5 and embryos were recovered after 20 h (E13.5). Fluorescence images were taken in the same condition. The genotype of each embryo is shown on the upper side of each panel. AID‐M indicates *CAG‐AID‐mCherry*. Scale bar, 1 mm. Quantitative data of fluorescence signals of each Type (without crossing with pMK411#19) are shown below pictures. (b) Fluorescence signals of mCherry in different organs dissected from E15.5 TG‐*CAG‐AID‐mCherry* (Type‐III) embryos. Bright‐field images are also shown for each organ.
**Figure S5.** Targeting strategy to establish CAG‐TIR1‐Flag line in *ROSA26* locus. The targeting vector was designed to insert *CAG‐TIR1(F74G)‐3xFlag* at the Cas9 target site (a). The knock‐in mouse was established via the ES‐mediated method. (b) Results of polymerase chain reaction (PCR) to detect 5′ and 3′ recombination. (c) PCR results of established mouse lines, *Rosa‐CAG‐TIR1* (RT) and *Rosa‐CAG‐AID‐mCherry* (RM).
**Figure S6.** Targeting strategy to establish *Rosa‐CAG‐AID‐mCherry* reporter line. The targeting vector was designed to insert *CAG‐AID‐mCherry* at the Cas9 target site of the *ROSA26* locus. The single‐stranded targeting vector was directly injected into a fertilized egg with sgRNA and Cas9 protein. The F0 mice were genotyped with specific primer sets to detect homologous recombination. #12 was used to establish the next generation.
**Figure S7.** Targeting strategy to establish AID‐DCP2 knock‐in mouse. The targeting vector was designed to insert an *AID‐tag* at the Cas9 target site in the exon1 of *Dcp2* in flame. The single‐stranded vector was directly injected into a fertilized egg with sgRNA and Cas9 protein. The F0 mice were genotyped with specific primer sets to detect homologous recombination. #13 was used to establish the next generation.
**Figure S8.** Targeting strategy to establish DCP2‐KO mouse via gene editing. Two sgRNAs were designed to remove exon‐2, which resulted in flame shift after splicing. Frozen sections were prepared from E9.5 wild‐type and DCP2‐null (KO) embryos and subjected to immunofluorescence staining against DDX6 antibody (green). Nuclear staining was conducted by Hoechst 33342 (blue). Scale bar, 20 μM.

**Table S1.** Guide RNA and primer information.

**Table S2.** Feature of mouse lines.
