# Supplementary material for: Establishment and characterization of mouse lines useful for endogenous protein degradation via an improved auxin‐inducible degron system (AID2)
Source: Dev Growth Differ. 2024 Sep 21;66(7):384–93. doi: 10.1111/dgd.12942 (PMC11482630; doi:10.1111/dgd.12942)
Supplement: Supplementary file 1 — Figure S1. Characterization of three independent transgenic mouse lines. (a) Adult mice of three independent lines, pMK411#13, #16, and #19, were dissected and the EGFP fluorescence of each organ was recorded. A C57BL/6J mouse was used as a negative control. The exposure and imaging conditions were the same. Scale bars, 1 mm. (b) The expression levels of transgenes in indicated organs were compared between pMK411#16 and #19 using anti‐TIR1, AID, and β‐actin antibodies. β‐actin expression was undetectable in the heart and quadriceps muscles. Figure S2. The integration sites of the transgene in pMK411#16 and #19. Both transgenes were inserted into chromosome 10 at different sites. Sequences around the insertion site and primer sets for genotyping are shown. Figure S3. Protein knockdown outside the body. (a) Parts of the adult brain and colon dissected from pMK411#13 were incubated in the medium with and without 5‐Ph‐IAA. The reporter EGFP signal was photographed every 1 h for up to 4 h. (b) EGFP reporter intensities of embryos were measured 2 h after 5‐Ph‐IAA injection at different concentrations (0–5.0 mg/kg) into pregnant MCH mothers crossed with pMK411#16 at E13.5. Scale bar, 1 mm. Figure S4. Characterization of transgenic mouse lines harboring CAG‐AID‐mCherry. (a) Three distinct TG lines (Type I–III) were established from one transgenic mouse (F0). Those lines were crossed with pMK411#19. The pregnant mother was injected with a single dose of 5‐Ph‐IAA (5 mg/kg) at E12.5 and embryos were recovered after 20 h (E13.5). Fluorescence images were taken in the same condition. The genotype of each embryo is shown on the upper side of each panel. AID‐M indicates CAG‐AID‐mCherry. Scale bar, 1 mm. Quantitative data of fluorescence signals of each Type (without crossing with pMK411#19) are shown below pictures. (b) Fluorescence signals of mCherry in different organs dissected from E15.5 TG‐CAG‐AID‐mCherry (Type‐III) embryos. Bright‐field images are also shown for each organ. [file DGD-66-384-s001.pdf]

Fig. S1

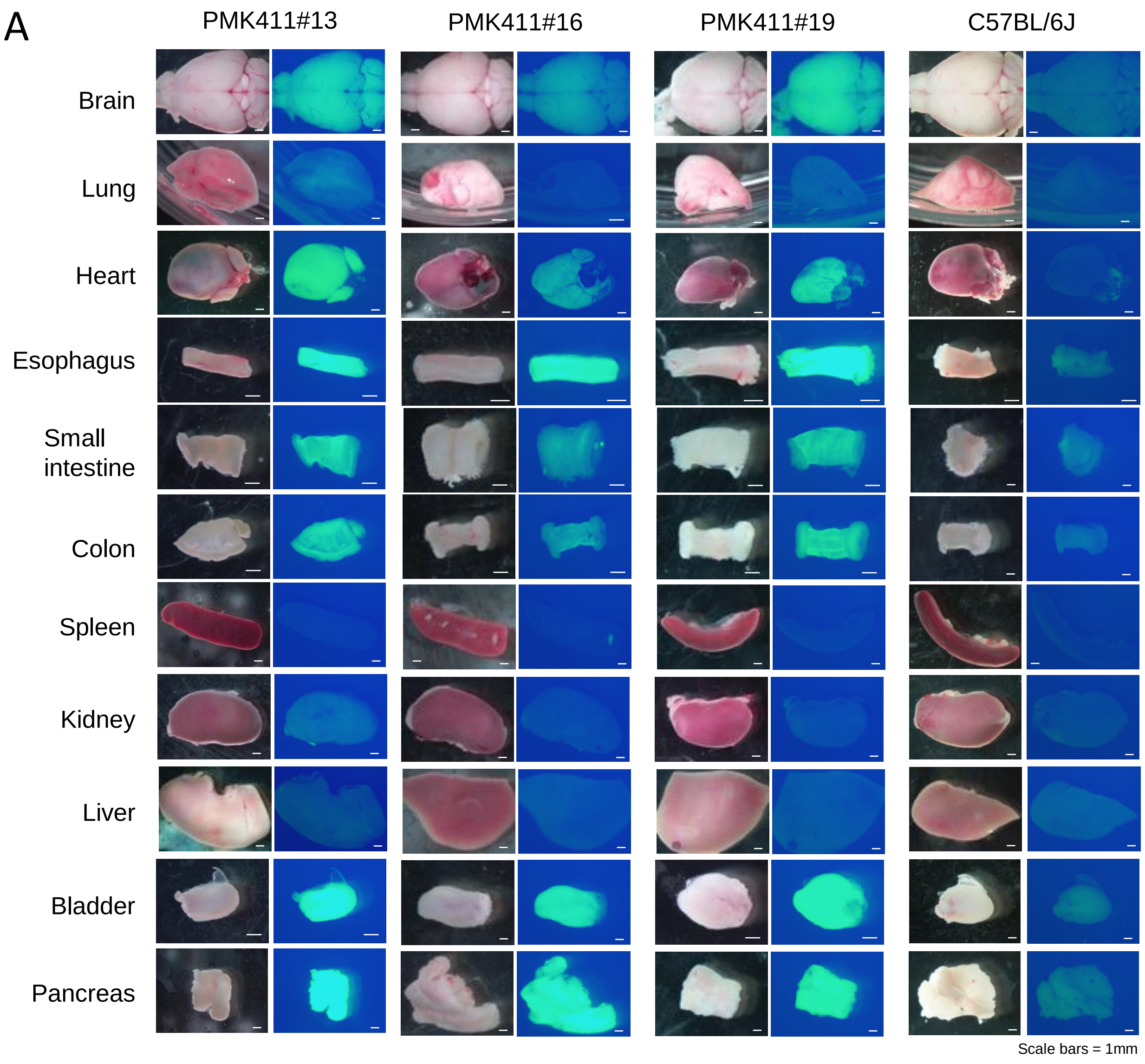
A
PMK411#13
PMK411#16
PMK411#19
C57BL/6J
Brain
Lung
Heart
Esophagus
Small intestine
Colon
Spleen
Kidney
Liver
Bladder
Pancreas
Scale bars = 1mm

Fig. S1B

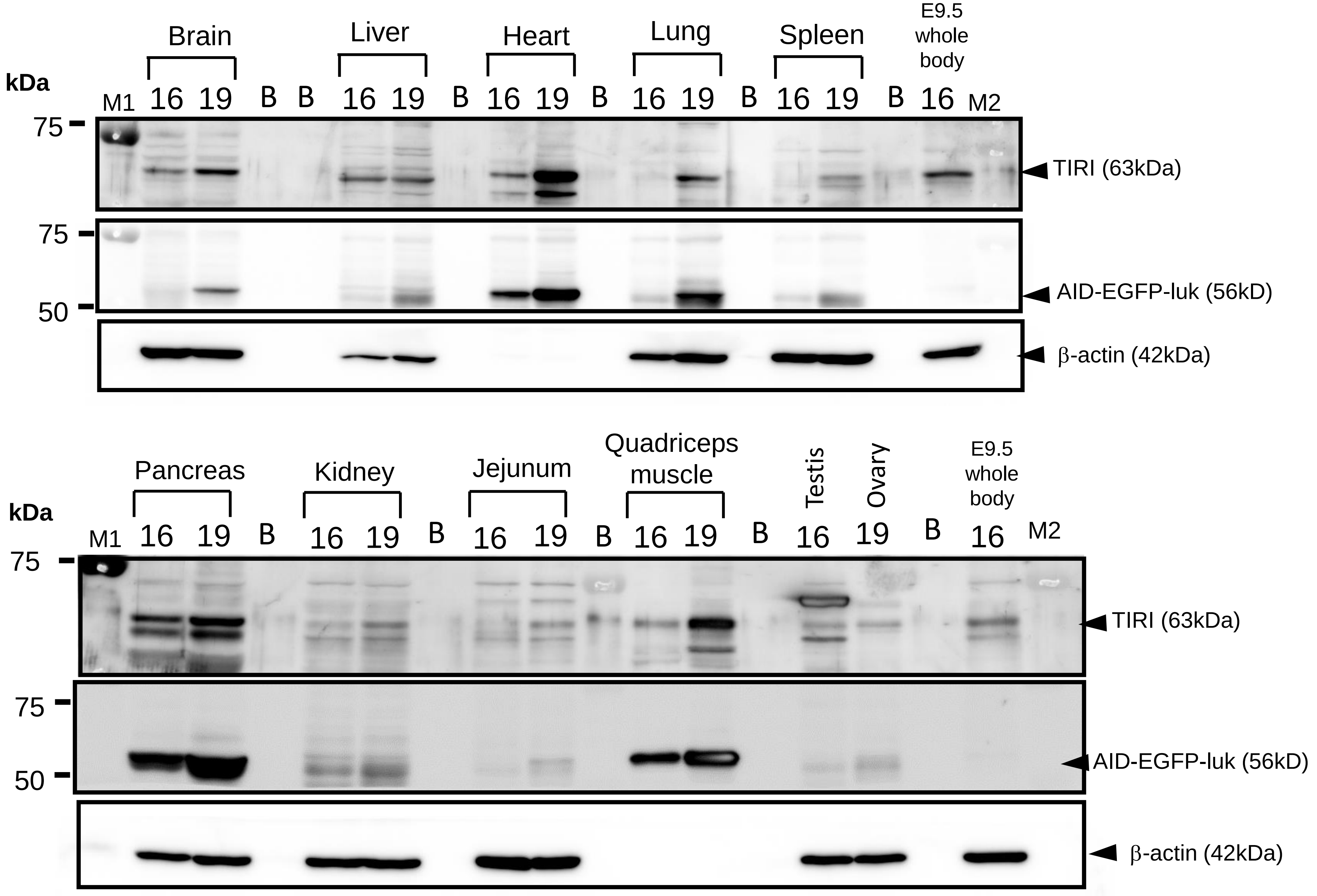

Brain
Liver
Heart
Lung
Spleen
E9.5 whole body
kDa
M1 16 19 B B 16 19 B 16 19 B 16 19 B 16 19 B 16 M2
75
75
50
TIRI (63kDa)
AID-EGFP-luk (56kD)
β-actin (42kDa)
Pancreas
Kidney
Jejunum
Quadriceps muscle
Testis
Ovary
E9.5 whole body
kDa
M1 16 19 B 16 19 B 16 19 B 16 19 B 16 19 B 16 M2
75
75
50
TIRI (63kDa)
AID-EGFP-luk (56kD)
β-actin (42kDa)

Fig. S2

A

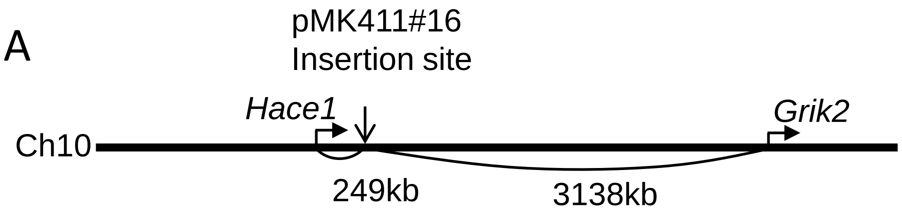

Sequence around insertion site:
Capital= Ch.10 genome, Lower=pMK411

TTCTAAACTTTCCATGCATATATCTAGAATTGCAGCTTTGCT
GTTATAGGCAACTGATCATTTTAAATGCAATGTATATTGCCA
AATTATTAATATTTCTCTTTTTAAAGGAAAGATGACATTTCC
TCCACATAATACTATCAAGAACATAAAATCTGAAAAACTCAC
AGTCAAAATCAAATTAAATAAATCTTTTCCTGGCAGAGATTA
AAGTCCAAAAAAAAAAAAAAaagatacattgatgagtttgga
caaaccacaactagaatgcagtgaaaaaaatgctttatttgt
gaaatttgtgatgctattgctttatttgtaaccattataagc
tgcaataaacaagttaacaacaacaattgcattcattttatg
tttcaggttcaggggaggtgtgggaggtttttttaaagcaag
taaaacctctacaaatgtggtatggc

Primer sets for genotyping

pMK411-16 IS WT Fw: 5'-GCAGCTTTGCTGTTATAGGCAAC-3'
pMK411-16 IS WT Rv: 5'- TCCACTCACAAAACGCTTAAACTT-3'
pMK411 HaeIII Rv1: 5'-TGCATTCTAGTTGTGGTTTGTCC-3'

B

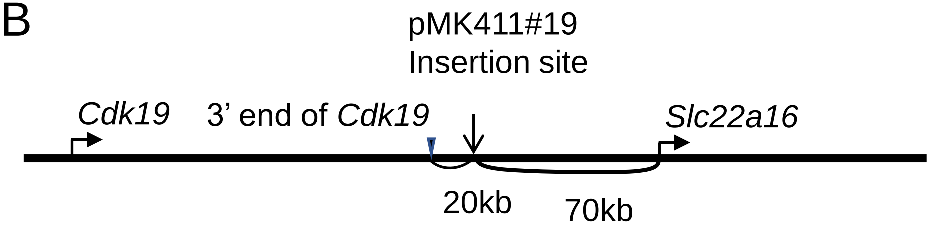

Sequence around insertion site:
Capital= Ch.10 genome, Lower=pMK411

GGCCCTATTGTGACCACTGGTCAACAAGGCAGGGCAAAGTCTGCCC
TTGCAGCAAAACCAGGAACCTCCCCACTTTTACGTGCTGAGAAGAG
TGTGTTTCGTATTGTGTAGATGGGGGTGGGGGCAGTTCCTTTAGAA
AAACAAAAAAACAGAACAAAAGCCAGACCGAGGAGAGAGATACTTA
TCTGGTCATCGGCTCCTGCCCCAGTGGTTTTAAATAACTCAAGCAG
TGGGCTTACTGTTCCCATTGCCTATTCCTCGTATCCCGCATCTTTC
TGTTACTGCTGACATTGTCCTTTTCACGAACTTCCTCTCACGCTAG
CCCTGAGCTCCTGTCGGGAATTCTCACTCATCAAGAGATGGCAGAC
TGTTAAAAAATATGTAGATATATTCAATAAATATTCTGTTCACATG
CACAGAGACATGGAAGAGCTTTTAATACAGAGCTTCAACTGGTTTG
GAACAGTTGTTATAGACAAATGCTGTGTAGTAGGCAGAGTGTTCAT
CGAAAATATGAGTTATTGTTGAGTGTGGTGGTGTATGCTTATAATG
TGAAATCTCGGGAGGGAAACTGACGCGttaagatacattgatgagt
ttggacaaaccacaactagaatgcagtgaaaaaaatgctttatttg
tgaaatttgtgatgctattgctttatttgtaaccattataagctgc
aataaacaagttaacaacaacaattgcattcattttatgtttcagg
ttcaggggaggtgtgggaggtttttttaaagcaagtaaaacctcta
caaatgtggtatggc

Primer sets for genotyping

pMK411-19 IS WT Fw2: 5'-ACATGCACAGAGACATGGAAGAG-3'
pMK411-19 IS WT Rv3: 5'- CACCCAGTCAGCACTCAGACTAAT-3'
pMK411 HaeIII Rv1: 5'-TGCATTCTAGTTGTGGTTTGTCC-3'

Fig. S3

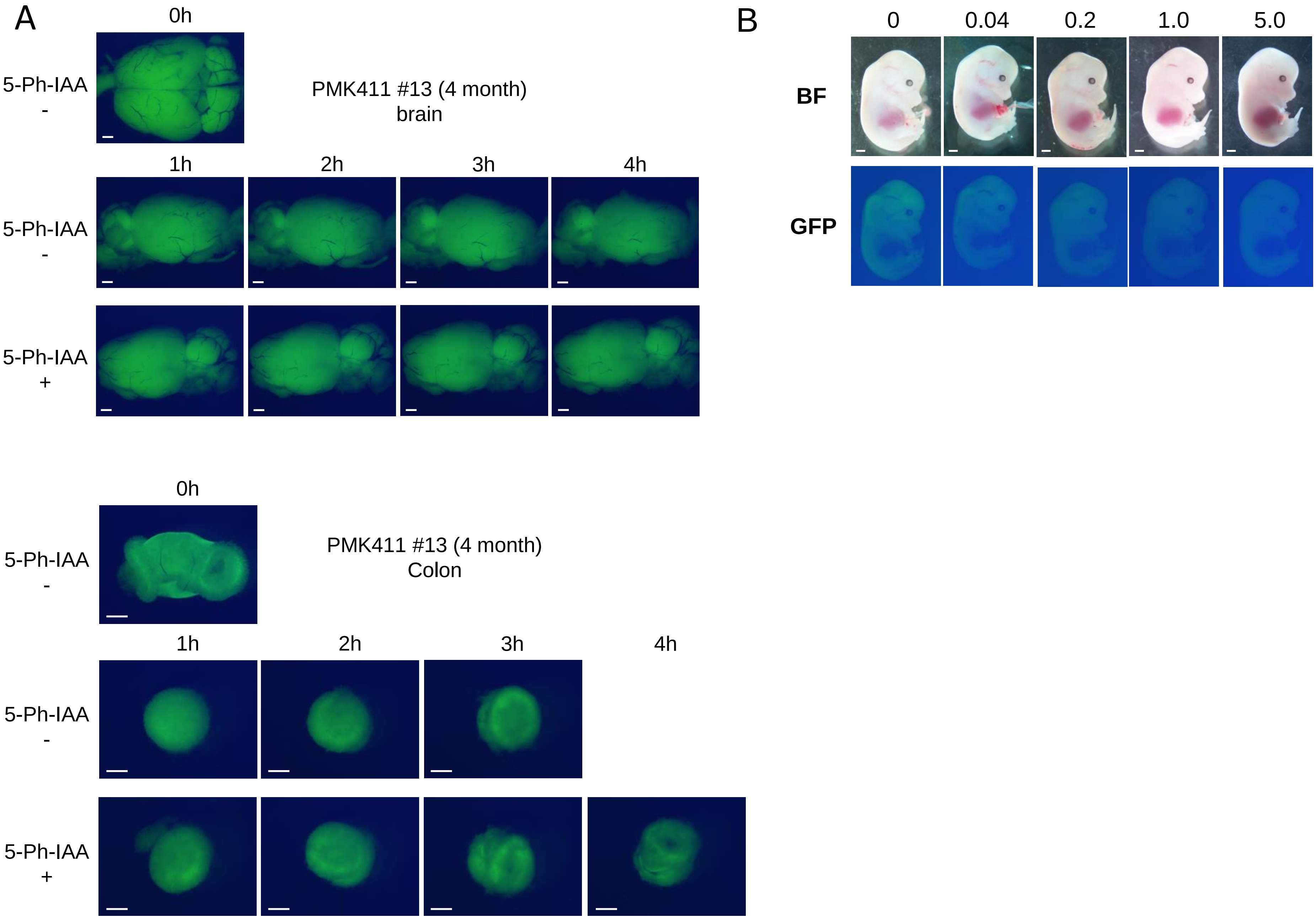
A
0h
5-Ph-IAA
-
PMK411 #13 (4 month)
brain
1h
2h
3h
4h
5-Ph-IAA
-
5-Ph-IAA
+
0h
5-Ph-IAA
-
PMK411 #13 (4 month)
Colon
1h
2h
3h
4h
5-Ph-IAA
-
5-Ph-IAA
+
B
0
0.04
0.2
1.0
5.0
BF
GFP

Fig. S4

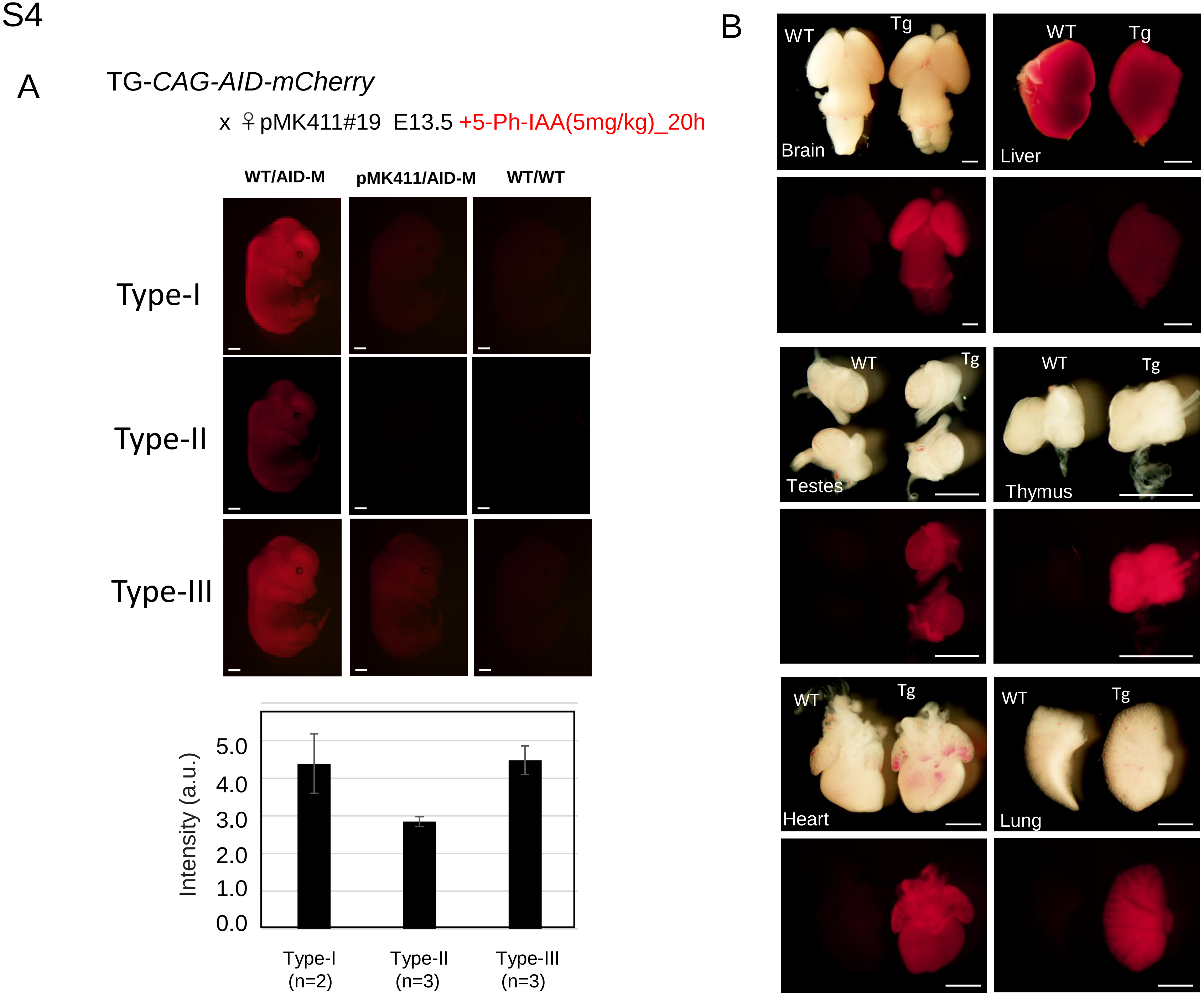
A
TG-CAG-AID-mCherry
x ♀pMK411#19 E13.5 +5-Ph-IAA(5mg/kg)_20h
WT/AID-M
pMK411/AID-M
WT/WT
Type-I
Type-II
Type-III
Intensity (a.u.)
5.0
4.0
3.0
2.0
1.0
0.0
Type-I (n=2)
Type-II (n=3)
Type-III (n=3)
B
WT
Tg
Brain
Liver
Testes
Thymus
Heart
Lung

Fig. S5

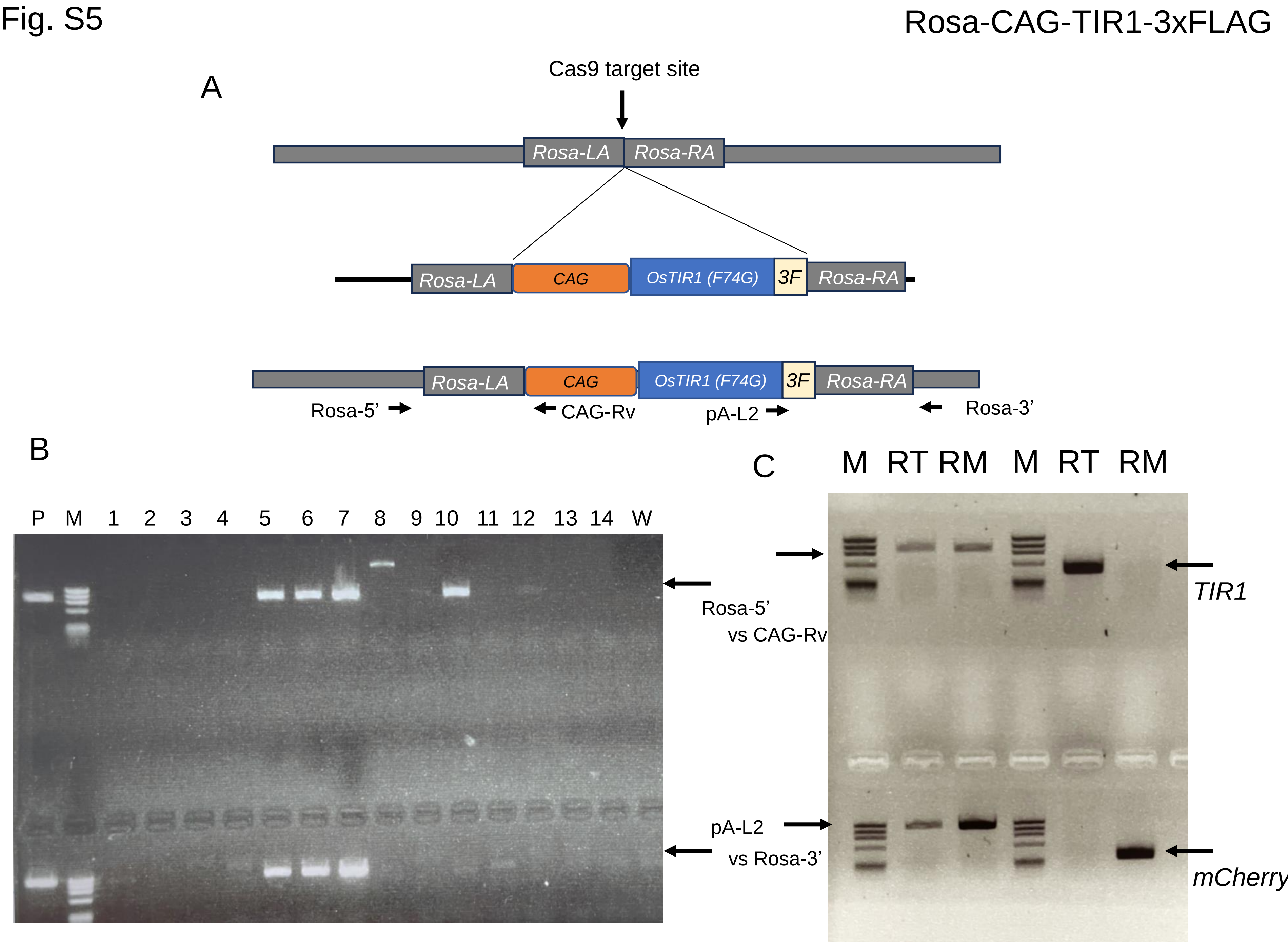

Rosa-CAG-TIR1-3xFLAG
A
Cas9 target site
Rosa-LA
Rosa-RA
Rosa-LA
CAG
OsTIR1 (F74G)
3F
Rosa-RA
Rosa-LA
CAG
OsTIR1 (F74G)
3F
Rosa-RA
Rosa-5’
CAG-Rv
pA-L2
Rosa-3’
B
P M 1 2 3 4 5 6 7 8 9 10 11 12 13 14 W
Rosa-5’ vs CAG-Rv
pA-L2 vs Rosa-3’
C
M RT RM M RT RM
TIR1
mCherry

Fig. S6

Rosa-CAG-AID-mCherry

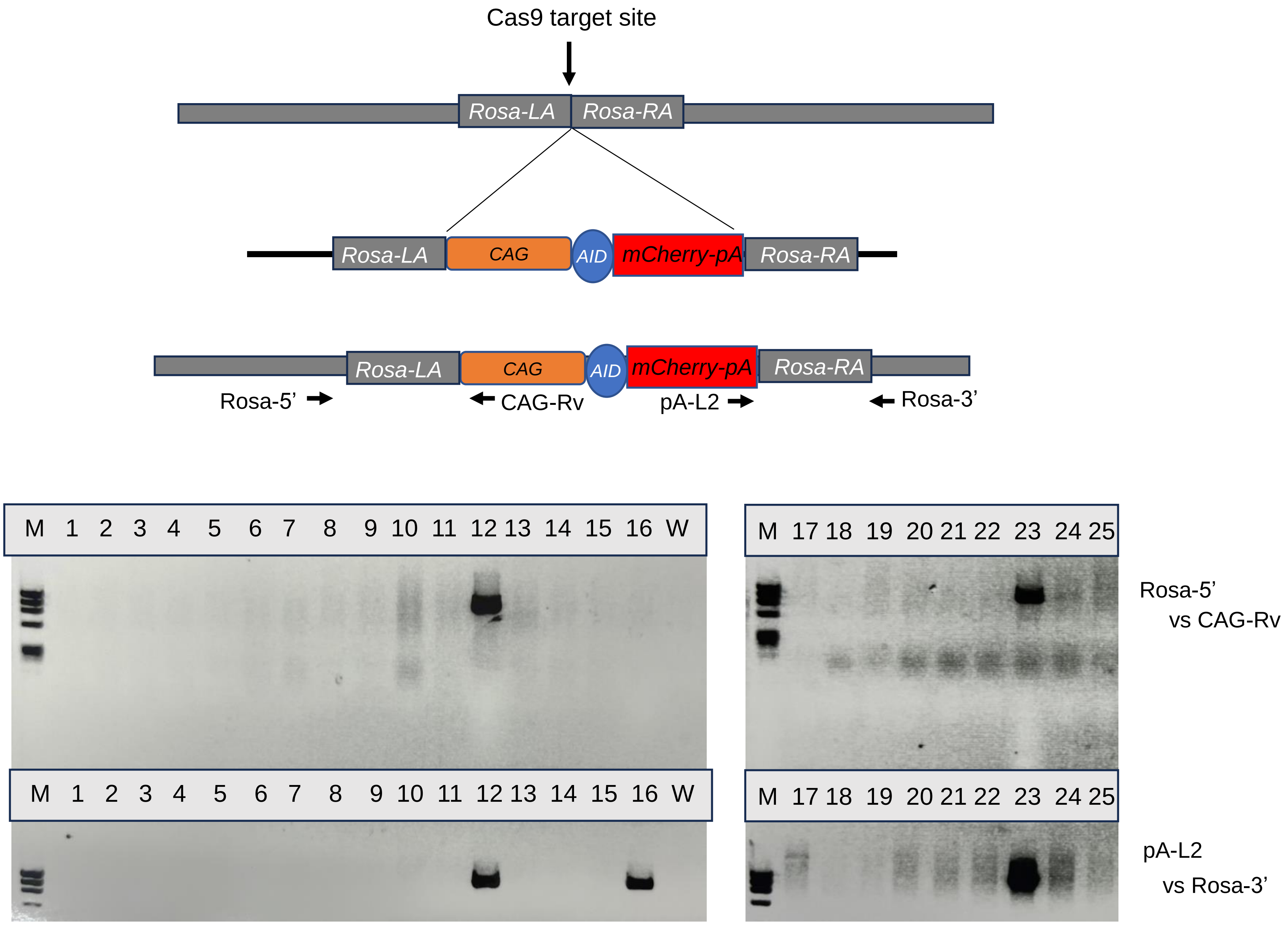

Fig. S7

AID-DCP2

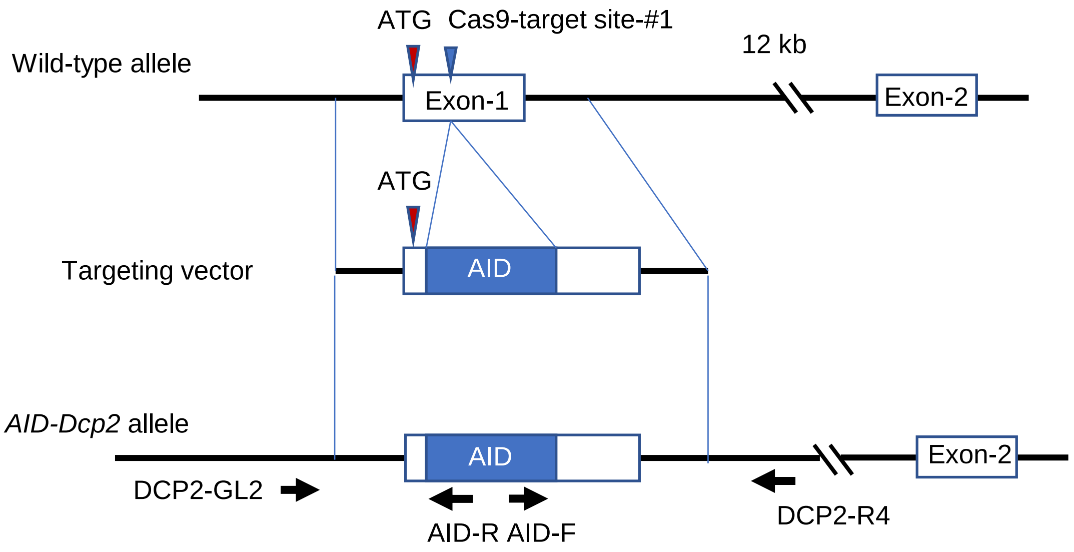

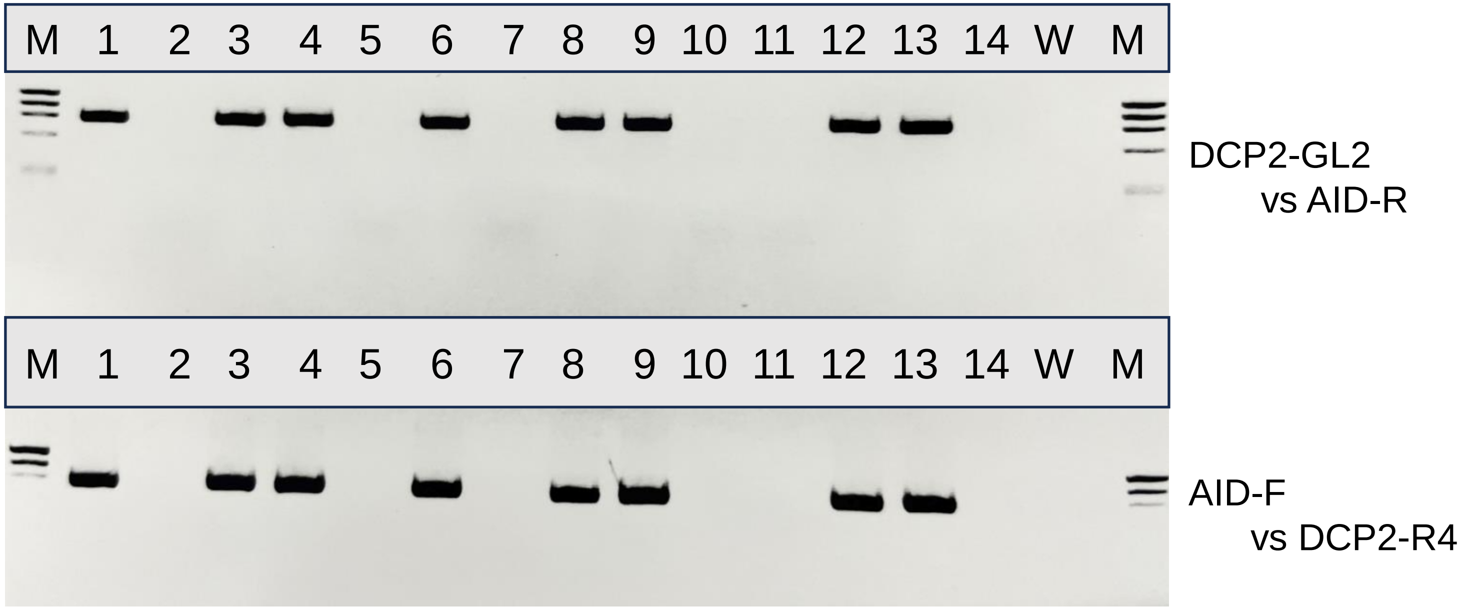

Fig. S8

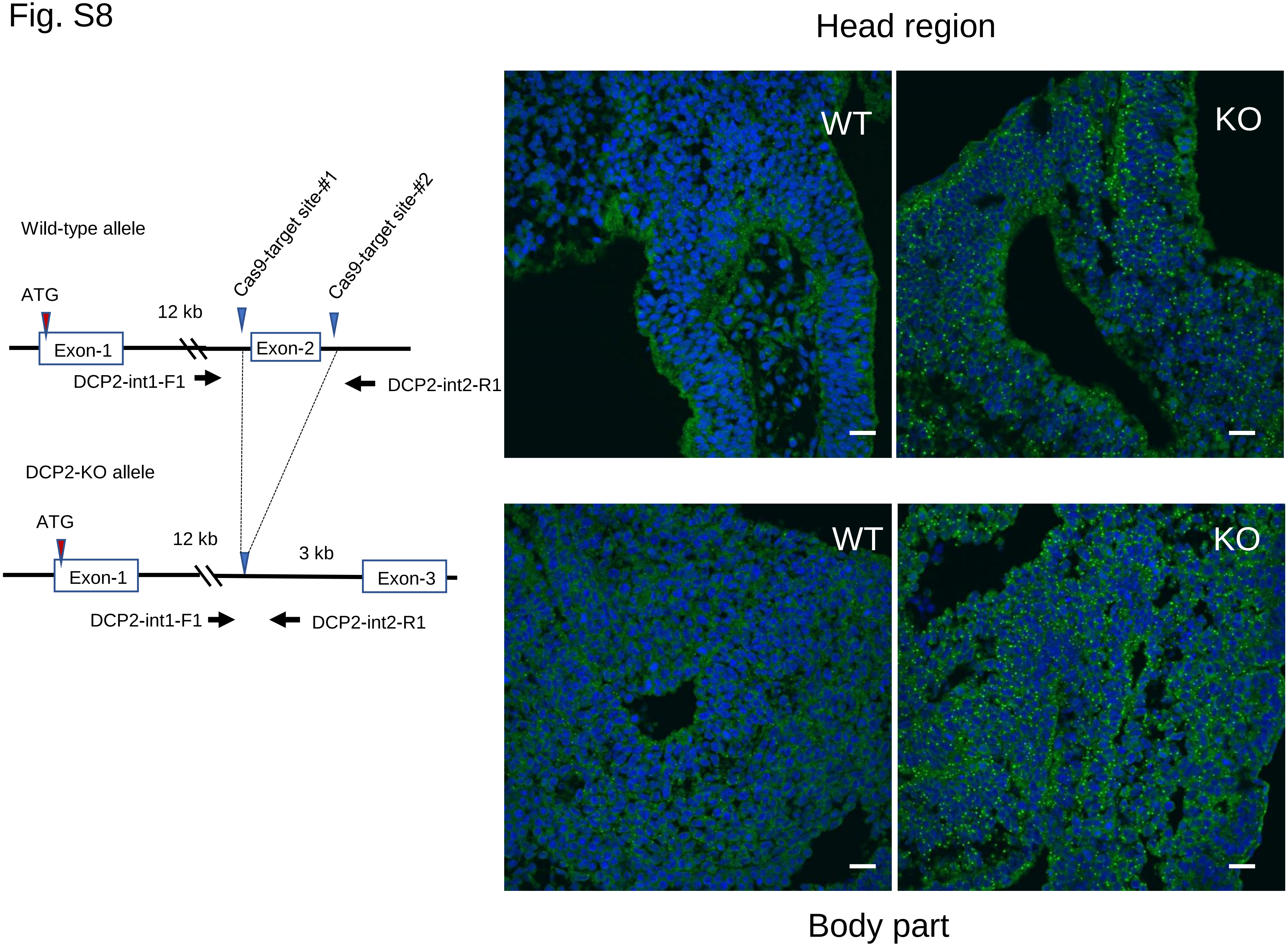
Head region
Wild-type allele
Cas9-target site-#1
Cas9-target site-#2
ATG
12 kb
Exon-1
Exon-2
DCP2-int1-F1
DCP2-int2-R1
DCP2-KO allele
ATG
12 kb
3 kb
Exon-1
Exon-3
DCP2-int1-F1
DCP2-int2-R1
WT
KO
WT
KO
Body part
